# Three-dimensional Evaluation of Gait: Kinetics, Kinematics, and Electromyographic in Patients with Mucopolysacharidosis Types IV and VI

**DOI:** 10.1055/s-0044-1786200

**Published:** 2024-07-22

**Authors:** Francisco Robson Queiroz Rego, Herison Franklin Viana de Oliveira, Epitácio Leite Rolim Filho

**Affiliations:** 1 Universidade Federal de Pernambuco, Recife, PE, Brasil

**Keywords:** electromyography, kinematics, kinetics, mucopolysaccharidoses, three-dimensional gait evaluation

## Abstract

**Objective** This study evaluated and determined, through instrumented three-dimensional (3D) gait analysis, the kinetic, kinematic, and electromyographic profile of patients with mucopolysaccharidosis (MPS) IV and VI.

**Methods** This crossectional study included 11 patients treated at a rare diseases reference service and evaluated in a movement analysis laboratory. We collected clinical, physical examination, and kinetic, kinematic, and electromyographic data using a 3D movement system, from June 2020 to January 2021.

**Results** There were 5 (45.5%) female patients, and 6 (54.5%) males. Furthermore, 9 (81.8%) subjects had MPS VI, and 2 (18.2%) had MPS IV. Their average age was 14.6 years. The average speed was 0.68 m/s (±0.21), and the stride length was 0.66 (±0.15). The most altered static angles were the hips' abduction-adduction, knees' range of movement, and foot's progression angle. Most cases presented a gait pattern of hip flexion-adduction and knee flexion. The gait profile scale (GPS) was 14.58 (±6.72) on the right side and 11.71 (±3.39) on the left. The gait deviation index (GDI) was 73.23 (±14.50) on the right side and 80.45 (±17.05) on the left. Muscle activity approximately followed the current model.

**Conclusion** The patients showed a decreased average speed and stride length. Most cases presented a gait pattern of hip flexion-adduction and knee flexion. Both GPS and GDI showed a significant deviation from normality.

## Introduction

Mucopolysaccharidoses (MPS) are rare genetic syndromes constituting the largest group of lysosomal storage diseases. Affected subjects present abnormal intracellular micromolecule degradation by lysosomal enzymes, leading to the intracellular accumulation of semi-degraded compounds called glycosaminoglycans (GAG). 1

Its general incidence is estimated as 1 in every 25,000 live births, varying according to country and ethnic origin. The most common MPS are the Morquio (MPS IV) and Hurler (MPS I) syndromes. 2 3 In Brazil, the most common subtypes are MPS II (Hunter syndrome) and VI (Maroteaux-Lami syndrome). 4

There are specific mutations in each type of MPS 2 5 resulting in a characteristic accumulation of GAGs and allowing MPS classification into seven types.

The clinical picture is quite diverse, involving multiple organs and systems, presenting different phenotypes and a range of functionality and severity; it can be fatal in some cases. 6 7 Limited ambulation and reduced resistance due to cardiopulmonary disease, joint stiffness, contractures, pain, and skeletal deformities are significant and progressive issues for many of these patients.

The diagnosis of MPS relies on GAG level, enzymatic activity, and genotype determination. 8 Chorionic villi and amniotic fluid cell samples establish prenatal diagnosis in fetuses. 2

Enzyme replacement therapy (ERT) and hematopoietic stem cell transplantation (HSCT) are the most common treatment options. Furthermore, several surgical techniques are used to treat musculoskeletal changes.

Few studies obtained appropriate functional measurements to monitor the MPS course and evaluate the impact of current therapeutic options. These studies highlight the difficulty in carrying out traditional performance tests in this population, including resistance and strength tests using treadmills, exercise bikes, and dynamometry. This partly occurs because MPS patients present reduced limb size and stature, joint diseases, limited strength, and a broad clinical spectrum. Assessing these patients is more effective using tests incorporating daily activities such as walking. 9 In these cases, an alternative is computerized gait analysis. 10

The importance of this tool motivated the development of indices to synthesize gait analysis data, facilitating its understanding. 11 Two of these data, the gait profile scale (GPS) and the gait deviation index (GDI) are global measures of gait variability and are sensitive to detect relevant changes in the deambulation of patients with orthopedic and neurological disorders. 12 13

The objective of the present study was to evaluate and determine, through instrumented three-dimensional (3D) gait analysis, the kinetic, kinematic, and electromyographic (EMG) profile of patients with MPS IV and VI in a reference service for rare diseases.

## Material and Methods

The Research Ethics Committee of the proposing Institution approved this cross-sectional, descriptive, and analytical study under number CAAE 40847720.5.0000.5208. The 3D gait analysis examination used the BTS-Gaitlab Hardware (BTS Bioengineering, Milan, Italy), and data analysis employed Excel (Microsoft Corp. Redmond, WA, USA) version 2301 and the Statistical Package Social Sciences (SPSS IBM Corp. Armonk, NY, USA) version 22. The study included 11 patients with MPS IV and VI evaluated from June 2020 to January 2021. The choice for both types of MPS occurred because affected subjects typically have better cognitive capacity, facilitating the test. Additionally, these are the most frequent MPS types monitored at the partner service.

The inclusion criteria were patients aged 4 years and older, diagnosed with MPS IV or VI, deambulatory, and treated at the partner service who agreed to participate in the study after invitation, with the patient or legal guardian having signed the informed consent form (ICF). Those with confirmation of other potential conditions, severe heart diseases, and cognitive, visual, or auditory deficits that compromised test application were excluded from the study.

**Fig. 1:**
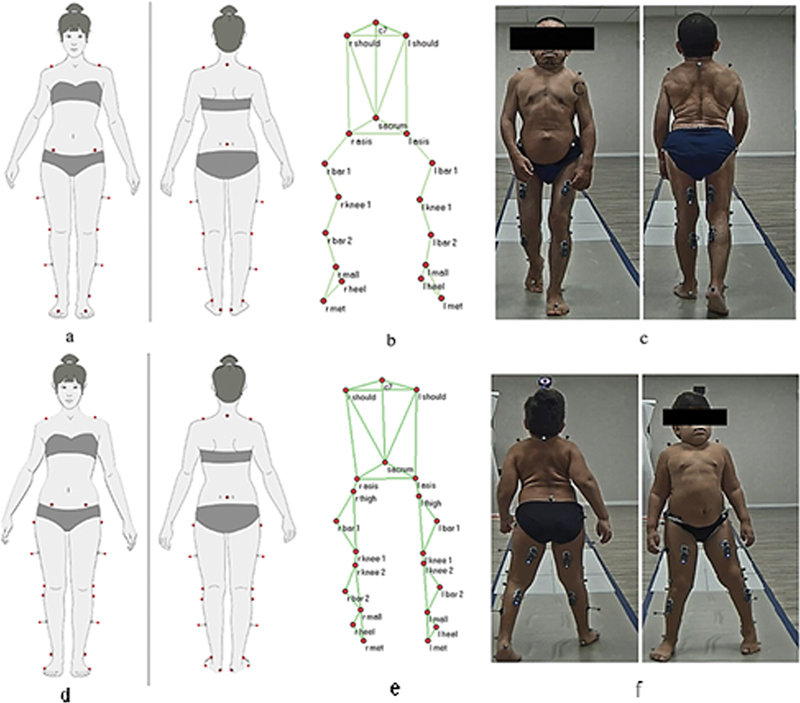
( **a** ) Front and back view of the modified Helen Hayes protocol. ( **b** ) Helen Hayes protocol markers placed on the model. ( **c** ) Patient performing gait analysis using the Helen Hayes protocol. ( **d** ) Front and back view of the Davis Protocol. ( **e** ) Davis protocol markers placed on the model. ( **f** ) Patient performing gait analysis using the Davis protocol.

We evaluated the following epidemiological variables: age, gender, disease classification, other conditions, and use of orthoses. The instrumented 3D gait analysis occurred per the following standardization. First, we collected anthropometric data, including weight, height, distance between the anterior superior iliac spines, pelvic depth, length of the lower limbs, and width of the knees and ankles. Next, we defined the BTS-Gaitlab protocol to carry out the test. For convenience and suitability for patients, especially concerning knee valgus deviations, we adopted the Helen Hayes or Modified Helen Hayes protocol for some subjects and the Davis protocol for others ( Fig. 1 ). Both protocols provide the same variables of interest.

Reflective markers for movement capturing were placed in anatomical locations defined by the chosen protocol. The same occurred for the EMG sensors, using the rectus femoris, semitendinosus, gastrocnemius, and anterior tibialis muscles as anatomical references.

After placing the markers and sensors, we asked the patients to perform two different tasks, for example, static and dynamic acquisition. For the static acquisition, patients remained in an orthostatic position for approximately 5 seconds on the force platforms. This protocol calculates joint angles and performs a 3D reconstruction of the patient to provide static data. For the dynamic acquisition, the patients walked naturally on the laboratory's track with six digital 3D force platforms to capture their ground reaction forces. Ten high-resolution, high-frequency infrared cameras captured information from the reflective markers to determine joint position and movement during gait. The EMG sensors captured the activity of the chosen muscles. Each patient performed approximately 18 repetitions. The data was recorded and transmitted to a computer via Bluetooth for processing and generation of a final report. On average, each complete exam lasted one hour.

The spatiotemporal variables of interest were cadence, speed (m/s), average speed (percentage of height/s), step length, stride length (m), stride length (percentage of height), width step, stance phase, swing phase, double stance, single stance, stride time, stance time and swing time. The hip assessment included inclination (pelvic tilt), obliquity, rotation, hip flexion-extension, and abduction-adduction angles. The range of motion evaluated knee (flexion-extension angle) and ankle (ankle dorsal plantar flexion) deviations. The progression angle determined foot deviations. Both GPS and GDI analyzed the general condition and gait quality.

We compared some analyzed variables with normative data to better understand potential movement compensation activities in different anatomical planes.

Regarding EMG data, we use its activation as a reference, i.e., whether the muscles were active or not (on-off) and during which phases of the gait cycle, as proposed by Sutherland. 13

The initial data analysis consisted of descriptive statistics, mean, standard deviation, absolute, and percentage frequency, as well as the validated gait analysis protocols integrated into BTS-Gaitlab. Statistical tests identified significant differences between the observed variables and the system's normality parameters. Non-parametric, Wilcoxon, and Mann-Whitney tests were used as an exact method for small samples. The 5% significance value indicated statistically significant correlation coefficients.

## Results

The present study included 11 patients with MPS ( Table 1 ), of which 5 (45.5%) were female and 6 (54.5%) were male. None of the patients used orthoses. Furthermore, 9 (81.8%) have MPS VI, and 2 (18.2%) IV. The patients' ages ranged from 4 to 43 years, with an average of 14.6.

**Table 1:** Sample characterization

Variable	N (%)
**Gender**	
Female	6 (54.5)
Male	5 (45.5)
**MPS**	
IV	2 (18.2)
VI	9 (81.8)
**Protocol type**	
Helen Hayes	6 (54.5)
Davis Heel	5 (45.5)
**Variable**	**Mean (SD)**
Weight (kg)	31.5 (13.1)
Height (cm)	113.6 (12.5)
Age (years)	14.6 (11.7)

**Abbreviation:** SD, standard deviation; MPS, mucopolysaccharidosis.

**Fig. 2:**
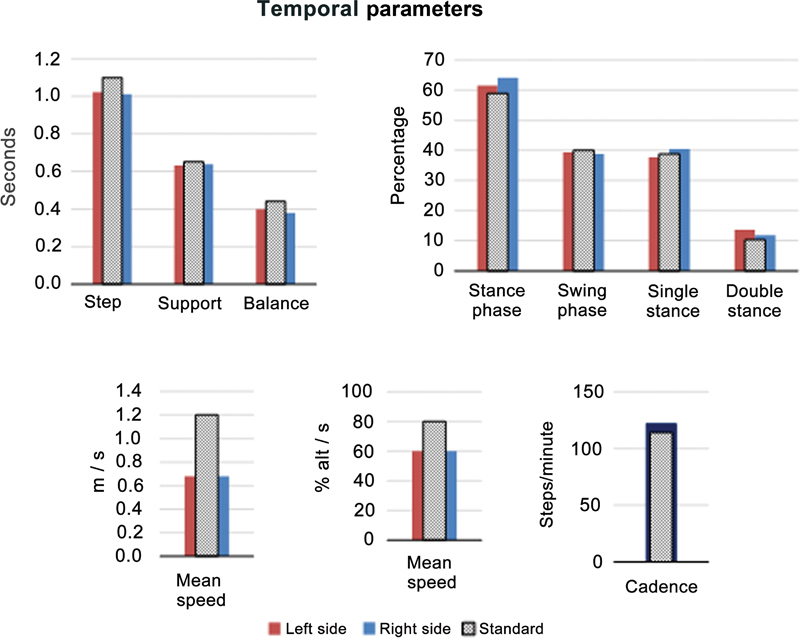
Mean values from each temporal parameter variable.

Regarding temporal parameters, the average speed (m/s) and average speed (percentage of height/s) were below the normality parameter. The average values of the other temporal parameters evaluated had little change or values within normal limits. As for spatial parameters, a mean stride length lower than the reference value is worth mentioning. Fig. 2 and Table 2 show this data.

**Table 2:** Mean (SD) of the variables observed at the three-dimensional gait analysis of the patients

	Standard value	Right limb	Left limb
**Temporal parameters**			
Stride time (s)	1.10 (0.09)	1.01* (0.17)	1.02* (0.16)
Stance time (s)	0.65 (0.07)	0.64 (0.11)	0.63 (0.13)
Swing time (s)	0.44 (0.05)	0.38* (0.06)	0.40* (0.04)
Stance phase (%)	58.98 (1.97)	64.11* (5.48)	61.62* (3.41)
Swing phase (%)	40.03 (3.56)	38.75 (6.49)	39.38 (3.11)
Single stance (%)	38.87 (2.57)	40.41 (5.72)	37.72 (4.02)
Double stance (%)	10.27 (3.09)	11.83 (2.35)	13.60* (4.40)
Mean speed (m/s)	1.20 (0.20)	0.68* (0.21)	
Mean speed (height %/s)	80.00 (5.00)	60.05* (19.98)	
Cadence (steps/min):	114.00 (4.20)	121.85* (18.02)	
**Spatial parameters ciais**			
Stride length (m)	1.36 (0.11)	0.66* (0.15)	0.67* (0.17)
Stride length (height %)	80.00 (10.00)	58.29* (14.20)	59.27* (15.32)
Step length (m)	0.62 (0.05)	0.35* (0.09)	0.33* (0.09)
Step width (m)	0.08 (0.05)	0.14* (0.07)	
**Static angles**			
Pelvic obliquity (°)	0.00 (1.00)	4.74* (12.60)	−0.46* (4.00)
Pelvic tilt (°)	10.00 (4.00)	13.85* (7.60)	13.85* (7.60)
Pelvic rotation (°)	0.00 (5.00)	1.23 (5.13)	−1.23 (5.13)
Hip abduction-adduction (°)	0.00 (3.00)	−6.51* (8.46)	−8.74* (7.64)
Hip flexion-extension (°)	10.00 (4.00)	17.25* (13.65)	20.32* (14.72)
Hip rotation (°)	0.00 (5.00)	3.45 (12.02)	3.05 (9.71)
Knee flexion-extension (°)	5.00 (5.00)	13.25 (17.17)	17.28* (14.31)
Ankle dorsal-plantar flexion (°)	0.00 (5.00)	−2.46* (38.64)	−6.61* (41.77)
Foot progression (°)	−10.00 (5.00)	−26.59* (15.40)	−16.55* (13.51)
**GPS**			
GPS (°)	< 7	14.85* (6.72)	11.71* (3.39)
**GVS**			
Pelvic obliquity (°)		4.50 (1.87)	4.94 (1.86)
Pelvic tilt (°)		7.86 (6.57)	7.85 (6.58)
Pelvic rotation (°)		6.06 (4.33)	6.34 (4.03)
Hip abduction-adduction (°)		8.22 (4.41)	9.57 (5.86)
Hip flexion-extension (°)		13.18 (5.87)	13.65 (6.89)
Hip rotation (°)		9.75 (5.36)	9.90 (4.74)
Knee flexion-extension (°)		15.38 (7.73)	12.99 (7.02)
Ankle dorsal-plantar flexion (°)		13.19 (13.65)	13.33 (11.87)
Foot progression (°)		14.04 (8.80)	10.64 (5.68)
**GDI**	> 100	73.23* (14.50)	80.45* (17.05)

**Abbreviation:** GDI, gait deviation index; GPS, gait profile scale; GVS, gait variable scores; SD, standard deviation. **Notes:** * p < 0.05 comparing with the standard value.

**Fig. 3:**
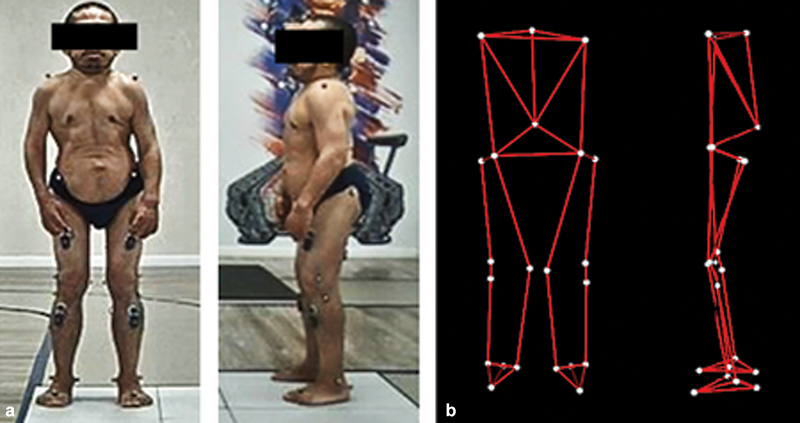
( **a** ) Patient during the static examination (standing) with Helen Hayes MM protocol in the gait laboratory; ( **b** ) The 3D reconstruction at BTS SMART-Clinic. Note the pattern of hip flexion.

In the evaluation of static angles ( Table 2 ), the hips showed an average flexion above the normal value ( Fig. 3 ). The range of motion of the knees also demonstrated important changes, reaching a maximum individual flexion value of 56.8° in the right knee and 48.9° in the left one. Most patients (54.5%) presented a gait pattern with increased hip flexion-adduction and knee flexion.

**Fig. 4:**
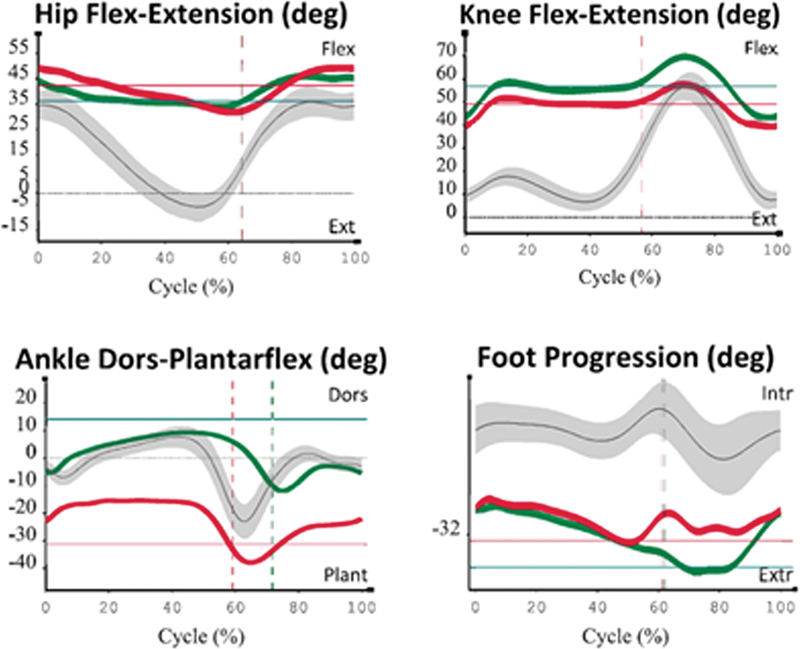
The BTS motion analysis graphs of different patients from the study. Note the deviation of the lower limb curves concerning the normality parameter in all images.

The mean GPS showed a significant deviation from the normality parameter. Regarding the gait variable score (GVS), hip flexion-extension and rotation, the range of movement of the knees and ankles, and the foot progression angle were the highest average values and were the major contributors to the elevation of the GPS value. All patients had a GDI below 100, and the lowest individual value was 59.67 ( Table 2 and Fig. 4 ).

The EMG data consisted of the activities of the rectus femoris, anterior tibiae, gastrocnemius (medial head), and semitendinosus muscles of 10 patients. One subject decided to undergo the examination without EMG.

Our results demonstrated intervals of muscular activity similar to the usual pattern. However, there was high variability between patients. Different activation modalities were captured and observed even in consecutive strides of a same subject. There were variations in the number of activations, onset time, and signal displacement. The muscles most activated during the gait cycle were the tibialis anterior (more active in 60% of patients) and the gastrocnemius. Muscle activation was asymmetric when comparing limbs from the same patient in 10% of cases.

## Discussion

Skeletal deformities are striking features present in different types of MPS. Angular defects, such as knee and ankle valgus, progressive hip dysplasia, and joint laxity/hypermobility are examples of changes resulting in gait dysfunction in these patients, 14 making walking and balance difficult and causing greater functional dependence in daily activities. 15

Matos et al. 16 studied 19 patients with MPS and showed that gait dysfunctions are the main complaints reported by this group and one of the major causes of functional disability in this population.

Human mobility represents a fundamental requirement for an adequate quality of life. The ability to walk is a general health indicator as it denotes autonomy. 17 Since walking is a basic requirement for many daily activities, gait analysis provides critical information about people's functional capacity.

The gait pattern of subjects has become an area of great interest. Gait analysis is widely used to diagnose diseases and evaluate therapeutic plans and the prognosis of several conditions. 18 Deviations from the typical gait pattern are frequent in neurological, muscular, and skeletal diseases. 19

Each MPS type has a wide range of clinical manifestations. 4 20 Furthermore, the same subtype can present several abnormal osteoarticular patterns and, therefore, gait patterns. The 3D assessment of these patients allows for a better understanding of the natural history of the disease in its different phenotypes.

The average speed and, mainly, stride length were below normal in our study, consistent with Salazar-Torres. 21 This finding indicates a higher metabolic demand for gait, as demonstrated by Kimoto et al. 22

Support time shows a fundamental correlation with stability and balance. High stance times represent a decrease in patients' ability to balance. 23 The average stance time in our study was normal, demonstrating that these patients have good stability during walking.

Another key aspect observed was the asymmetry between the values obtained for each member. The parameters with the most differences between limbs during walking were the knees' range of movement and the foot's progression angle. Asymmetry was also noted in temporal values and, to a lesser extent, in EMG data. It is known that the greater the asymmetry of the lower limbs during walking, the greater the energy consumption. 24

Most of our patients presented a pattern of flexion and adduction of the hips and flexion of the knees. Changes in these joints are well described in both the Maroteaux-Lamy and Morquio syndromes. It is known that, especially in the hips and knees of these patients, there may be impairment from the first years of life. 25

The GPS and GDI indices represent a summary of the general gait quality, facilitating the comparison between pathological and normal deambulation. Massad et al. 26 described GDI as a reliable measure for gait assessment even in a single session. In our study, both these score values showed an important deviation from normality.

The EMG data demonstrated intervals of muscular activity approximately following the current model. However, it is worth highlighting the high variability of this tests' patterns between patients, consistent with Agostini et al. 27 A limiting factor in the evaluation of these data in our study was that 54.5% of the patients were children, of which 45.5% ranged from 4 to 8-years-old. Although subjects from this age group have mature deambulation, Granata et al. 28 demonstrated that intrasession EMG variability in children aged 6 to 8 years is twice as high as in adults. Agostini et al. 27 stated that this exam's activity can vary drastically between children, with less than 50% showing a similar pattern. The large variability in children may indicate more responsive stabilization control than in adults. 27

A study by Fleming et al. 29 evaluated the level of functionality in 15 children with MPS using the International Classification of Functioning, Disability, and Health. These authors reported mild impairment of bodily functions and severe impairment of joint mobility and gait, which is in line with our study.

The limitation of this study was the small number of subjects. In genetic storage syndromes, involving different systems, research must deal with the high variability between patients. As such, these studies must include a large number of subjects. Therefore, further studies are required with a larger sample of patients.

## Conclusion

Patients presented a decreased average speed and stride length. Most cases presented a gait pattern of flexion and adduction of the hips and flexion of the knees. Both GPS and GDI showed an important deviation from normality. The analysis of EMG data revealed intervals of muscular activity approximately following the current model, highlighting the high variability of muscle activation patterns between patients.
