# Supplementary material for: Three-dimensional Evaluation of Gait: Kinetics, Kinematics, and Electromyographic in Patients with Mucopolysacharidosis Types IV and VI
Source: Rev Bras Ortop (Sao Paulo). 2024 Jul 22;59(5):e737–44. doi: 10.1055/s-0044-1786200 (PMC11624931; doi:10.1055/s-0044-1786200)
Supplement: Related texts

# Research article: Avaliação tridimensional da marcha: Cinética, cinemática e eletromiográfica em pacientes com mucopolissacaridose tipos IV e VI

**Keywords:** avaliação tridimensional da marcha, cinemática, cinética, eletromiografia, mucopolissacaridoses

## Abstract

**Objetivo** Avaliar e determinar, através de análise tridimensional (3D) instrumentada da marcha, o perfil cinético, cinemático e eletromiográfico de pacientes com mucopolissacaridose (MPS) IV e VI.

**Métodos** Foi realizado um estudo transversal com 11 pacientes atendidos em um serviço de referência em doenças raras, avaliados em laboratório de análise do movimento. Foram coletados dados clínicos, do exame físico e dados cinéticos, cinemáticos e eletromiográficos através de sistema 3D de movimento, entre junho de 2020 e janeiro de 2021.

**Resultados** Havia 5 pacientes (45,5%) do gênero feminino e 6 (54,5%) do masculino. Adicionalmente, 9 (81,8%) tinham MPS VI e 2 (18,2%) IV. A idade média foi de 14,6 anos. A velocidade média (m/s) foi de 0,68 (±0,21) e o comprimento da passada de 0,66 (±0,15). Os ângulos estáticos mais alterados foram a abdução-adução dos quadris, o arco de movimento dos joelhos e o ângulo de progressão do pé. Um padrão de marcha em flexão e adução dos quadris e flexão dos joelhos foi identificado na maioria dos casos. O *Gait Profile Score* (GPS) foi de 14,58 (±6,72) à direita e 11,71(±3,39) à esquerda. O *Gait Deviation Index* (GDI) foi de 73,23 (±14,50) à direita e de 80,45 (±17,05) à esquerda. A atividade muscular seguiu aproximadamente o modelo corrente.

**Conclusão** Os pacientes apresentaram uma velocidade média e comprimento da passada diminuídos. Um padrão de marcha em flexão e adução dos quadris e flexão dos joelhos foi identificado na maioria dos casos. Tanto GPS quanto GDI mostraram desvio importante da normalidade.

## Introdução

As mucopolissacaridoses (MPS) são síndromes genéticas raras que constituem o maior grupo das doenças de armazenamento lisossômico. A degradação intracelular de micromoléculas por enzimas lisossomais é anormal nesta população, levando ao acúmulo intracelular de compostos semidegradados, as glicosaminoglicanas (GAG). 1

Sua incidência geral é estimada em 1 a cada 25.000 nascidos vivos, variando em cada país e origem étnica, sendo as mais comuns as síndromes de Morquio (MPS IV) e Hurler (MPS I). 2 3 No Brasil, os subtipos mais comuns encontrados são MPS II (síndrome de Hunter) e VI (síndrome de Maroteaux-Lami). 4

Mutações específicas são descritas individualmente para cada tipo de MPS 2 5 levando a um acúmulo característico de GAGs, permitindo a classificação das MPS em sete tipos.

O quadro clínico é bastante diversificado, com acometimento de múltiplos órgãos e sistemas, apresentando diferentes fenótipos e graus variados de funcionalidade e gravidade, podendo ser fatal em alguns casos. 6 7 Deambulação limitada e resistência reduzida, causadas por doença cardiopulmonar, rigidez articular, contraturas, dor e deformidades esqueléticas, são problemas significativos e progressivos para muitos destes pacientes.

A determinação do nível de GAGs, bem como a atividade enzimática e genotipagem são fundamentais para o diagnóstico das MPS. 8 Amostragens de vilosidades coriônicas e células do líquido amniótico são utilizados para estabelecer o diagnóstico pré-natal nos fetos. 2

A terapia de reposição enzimática (TRE) e o transplante de células-tronco hematopoiéticas (TCTH) são as opções de tratamento mais utilizadas. Além disso, diversas técnicas cirúrgicas são empregadas para tratamento das alterações musculoesqueléticas.

Poucos estudos obtiveram medidas funcionais apropriadas para monitorar o curso das MPS e avaliar o impacto das opções terapêuticas atualmente aplicadas. Estes estudos evidenciam a grande dificuldade para realização de testes de desempenho tradicionais nesta população, por exemplo os testes de resistência e de força em esteiras, bicicletas ergométricas e dinamometria. Isto se deve, dentre outros, ao tamanho reduzido dos membros e estatura, doenças articulares, força limitada e amplo espectro clínico das MPS. Estes pacientes podem ser avaliados de uma forma mais eficaz utilizando-se testes que incorporam atividades exigidas na vida diária, como o ato de caminhar. 9 Uma alternativa nestes casos é a análise computadorizada da marcha. 10

A importância desta ferramenta motivou o desenvolvimento de índices que podem sintetizar os dados da análise de marcha, facilitando seu entendimento. 11 Dois destes dados, a pontuação do perfil da marcha *Gait Profile Scale* , (GPS) e o índice de desvio da marcha *Gait Deviation Index* (GDI) são medidas globais de variabilidade da marcha, sendo sensíveis para detectar alterações relevantes na deambulação de pacientes com distúrbios ortopédicos e neurológicos. 12 13

O objetivo do presente estudo foi avaliar e determinar, através da análise tridimensional (3D) instrumentada da marcha o perfil cinético, cinemático e eletromiográfico de pacientes com MPS IV e VI em um serviço de referência em doenças raras.

## Material e Métodos

O presente estudo é um corte transversal, descritivo e analítico, aprovado pelo comitê de Ética em Pesquisa da Instituição proponente sob o número CAE 40847720.5.0000.5208. Para o exame de análise 3D da marcha foi utilizado o Hardware BTS-Gaitlab (BTS Bioengineering, Milão, Itália) e para a análise dos dados foram usados os softwares Excel (Microsoft Corp., Redmond, WA, EUA) versão 2301 e o *Statistical Package Social Sciences* (SPSS, IBM Corp., Armonk, Nova Iorque, EUA) versão 22. O Estudo incluiu 11 pacientes com MPS tipos IV e VI avaliados no período entre junho de 2020 e janeiro de 2021. A escolha por ambos os tipos de MPS atribuiu-se ao fato de os mesmos terem caracteristicamente uma melhor capacidade cognitiva, facilitando a coleta do exame, e também por serem os tipos mais frequentes acompanhados no serviço parceiro.

Os critérios de inclusão foram pacientes com idade a partir de 4 anos, com diagnóstico de MPS IV ou VI, deambuladores, atendidos no serviço parceiro, que aceitaram participar do estudo após convite e terem concordado e assinado (o paciente ou responsável legal) o Termo de Consentimento Livre e Esclarecido (TCLE). Foram excluídos do estudo os pacientes com confirmação de outras possíveis etiologias, pacientes portadores de doença cardíaca grave, déficit cognitivo, visual ou auditivo que comprometessem a aplicação do exame.

As seguintes variáveis epidemiológicas foram avaliadas: idade, gênero, classificação da doença, outras patologias e uso de órteses. A análise 3D instrumentada da marcha seguiu a seguinte padronização: inicialmente foi realizada a coleta dos dados antropométricos - peso, altura, distância entre as espinhas ilíacas anterossuperiores, profundidade pélvica, comprimento dos membros inferiores, largura dos joelhos e dos tornozelos. Após esta etapa, foi definido o protocolo do BTS-Gaitlab que seria utilizado para realização do exame. Por conveniência e adequação aos pacientes, especialmente em relação a desvios em valgo dos joelhos, foi adotado para uns o protocolo Helen Hayes ou Modified Helen Hayes e para outros o protocolo Davis ( Fig. 1 ), ambos fornecendo as mesmas variáveis de interesse.

**Fig. 1:** ( **a** ) Visão frontal e posterior do protocolo modified Helen Hayes. ( **b** ) Marcadores do protocolo Helen Hayes colocados no modelo. ( **c** ) Paciente realizando análise de marcha utilizando protocolo Helen Hayes. ( **d** ) Visão frontal e posterior do protocolo Davis. ( **e** ) Marcadores do protocolo Davis colocados no modelo. ( **f** ) Paciente realizando análise de marcha utilizando protocolo Davis.

Os marcadores reflexivos para captura do movimento foram posicionados em locais anatômicos definidos pelo protocolo escolhido, assim como os sensores para captação da eletromiografia (EMG), tendo como referências anatômicas os músculos retos femorais, semitendinosos, gastrocnêmios e tibiais anteriores.

Após colocação dos marcadores e sensores, os pacientes foram solicitados a realizar duas tarefas diferentes: tomada estática e tomada dinâmica. Para tomada estática os pacientes foram orientados a manter-se em posição ortostática por cerca de 5 segundos sobre as plataformas de força. Este protocolo calcula os ângulos articulares e realiza uma reconstrução 3D do paciente, fornecendo seus dados estáticos. Para a tomada dinâmica, os pacientes foram orientados a caminhar naturalmente na pista presente do laboratório, na qual estavam acopladas seis plataformas de força 3D digitais, que capturavam as forças de reação ao solo dos pacientes. Os marcadores reflexivos foram captados por 10 câmeras infravermelhas de alta resolução e frequência, fornecendo informações sobre a posição e o movimento das articulações durante a marcha. Os sensores de EMG captaram a atividade dos músculos escolhidos. Foram feitas cerca de 18 repetições para cada paciente. Os dados foram registrados e transmitidos para um computador via bluetooth, onde foram processados, gerando um relatório final. Cada exame completo durou, em média, uma hora.

Como variáveis espaço-temporais de interesse foram consideradas: cadência, velocidade (m/s), velocidade média (percentual da altura/s), comprimento do passo, comprimento da passada (m), comprimento da passada (percentual da altura), largura do passo, fase de apoio, fase de balanço, duplo apoio, apoio simples, tempo de passada, tempo de apoio e tempo de balanço. Para a avaliação do quadril foram considerados os índices de inclinação ( *tilt* pélvico), obliquidade, rotação e os ângulos de flexão-extensão e abdução-adução do quadril. O arco de movimento foi utilizado para avaliar os desvios do joelho (ângulo de flexo-extensão) e do tornozelo (dorsi-plantiflexão do tornozelo). Para avaliação dos desvios do pé foi utilizado o ângulo de progressão. Foram utilizados o GPS e o GDI como índices para análise da patologia geral e qualidade da marcha.

Algumas das variáveis analisadas foram comparadas com dados normativos, visando obter uma melhor compreensão das possíveis atividades de compensação do movimento nos diferentes planos anatômicos.

Sobre os dados da EMG, usamos como referência sua ativação. Ou seja, se a musculatura estava ativa ou não (on-off) e em quais fases do ciclo da marcha, conforme proposto por Sutherland. 13

A análise inicial dos dados obtidos dos pacientes foi realizada pelo cálculo de estatísticas descritivas, média, desvio-padrão, frequência absoluta e percentual, assim como os protocolos validados de análise de marcha integrados no BTS Gaitlab. Foram usados testes estatísticos para identificar diferenças significativas entre os valores observados nas variáveis estudadas e os parâmetros de normalidade do sistema. Foram utilizados testes não-paramétricos, Wilcoxon e Mann-Whitney, método exato para amostras pequenas. Foi considerado o valor de significância igual a 5%, usado para indicar os coeficientes de correlação estatisticamente significativos.

## Resultados

O presente estudo inclui 11 pacientes com MPS ( Tabela 1 ), dos quais 5 (45,5%) são do gênero feminino e 6 (54,5%) do masculino. Nenhum dos pacientes fazia uso de órteses. Além disso, 9 (81,8%) são portadores de MPS VI e 2 (18,2%) são portadores de MPS IV. A idade dos pacientes variou de 4 a 43 anos, com média de 14,6.

**Tabela 1:** Caracterização da amostra

Variável	N (%)
**Gênero**	
Feminino	6 (54,5)
Masculino	5 (45,5)
**MPS**	
IV	2 (18,2)
VI	9 (81,8)
**Tipo de protocolo**	
Helen Hayes	6 (54,5)
Davis Heel	5 (45,5)
**Variável**	**Média (DP)**
Peso (kg)	31,5 (13,1)
Altura (cm)	113,6 (12,5)
Idade (anos)	14,6 (11,7)

**Abreviações:** DP, desvio padrão; MPS, mucopolissacaridoses.

Com relação aos parâmetros temporais, a velocidade média (m/s) e a velocidade média (percentual da altura/s) estavam abaixo do parâmetro de normalidade. Os valores médios dos demais parâmetros temporais avaliados tiveram pouca alteração ou valores dentro dos padrões da normalidade. Na avaliação dos parâmetros espaciais, chama atenção um comprimento da passada médio inferior ao valor de referência. Os dados estão representados na Fig. 2 e na Tabela 2 .

**Fig. 2:** Valores médios observados para cada variável dos parâmetros temporais.

**Tabela 2:** Média (DP) dos valores observados nas variáveis dos exames de análise tridimensional da marcha dos pacientes

	Valor padrão	Membro direito	Membro esquerdo
**Parâmetros Temporais**			
Tempo de passada (s):	1,10 (0,09)	1,01* (0,17)	1,02* (0,16)
Tempo de apoio (s):	0,65 (0,07)	0,64 (0,11)	0,63 (0,13)
Tempo de balanço(s):	0,44 (0,05)	0,38* (0,06)	0,40* (0,04)
Fase de apoio (%):	58,98 (1,97)	64,11* (5,48)	61,62* (3,41)
Fase de balanço (%):	40,03 (3,56)	38,75 (6,49)	39,38 (3,11)
Fase de apoio simples (%)	38,87 (2,57)	40,41 (5,72)	37,72 (4,02)
Fase de duplo apoio (%):	10,27 (3,09)	11,83 (2,35)	13,60* (4,40)
Velocidade média (m/s):	1,20 (0,20)	0,68* (0,21)	
Velocidade média (%altura/s):	80,00 (5,00)	60,05* (19,98)	
Cadência (steps/min):	114,00 (4,20)	121,85* (18,02)	
**Parâmetros Espaciais**			
Comprimento da passada (m):	1.36 (0.11)	0,66* (0,15)	0,67* (0,17)
Comprimento da passada (%alt.):	80,00 (10,00)	58,29* (14,20)	59,27* (15,32)
Comprimento do passo (m):	0.62 (0.05)	0,35* (0,09)	0,33* (0,09)
Largura do Passo (m):	0.08 (0.05)	0,14* (0,07)	
**Ângulos estáticos**			
Obliquidade pélvica (graus):	0,00 (1,00)	4,74* (12,60)	−0,46* (4,00)
Tilt pélvico (graus):	10,00 (4,00)	13,85* (7,60)	13,85* (7,60)
Rotação pélvica (graus):	0,00 (5,00)	1,23 (5,13)	−1,23 (5,13)
Ab-adução do quadril (°):	0,00 (3,00)	−6,51* (8,46)	−8,74* (7,64)
Flexo-extensão do quadril (°):	10,00 (4,00)	17,25* (13,65)	20,32* (14,72)
Rotação do quadril (°):	0,00 (5,00)	3,45 (12,02)	3,05 (9,71)
Flexo-extensão do joelho (°):	5,00 (5,00)	13,25 (17,17)	17,28* (14,31)
Dorso-flexão plantar do tornozelo (°):	0,00 (5,00)	−2,46* (38,64)	−6,61* (41,77)
Progressão do pé (°):	−10,00 (5,00)	−26,59* (15,40)	−16,55* (13,51)
**GPS**			
Obliquidade pélvica (graus):	< 7	14,85* (6,72)	11,71* (3,39)
**GVS**			
Obliquidade pélvica (graus):		4,50 (1,87)	4,94 (1,86)
Tilt pélvico (graus):		7,86 (6,57)	7,85 (6,58)
Rotação pélvica (graus):		6,06 (4,33)	6,34 (4,03)
Ab-adução do quadril (°):		8,22 (4,41)	9,57 (5,86)
Flexo-extensão do quadril (°):		13,18 (5,87)	13,65 (6,89)
Rotação do quadril (°):		9,75 (5,36)	9,90 (4,74)
Flexo-extensão do joelho (°):		15,38 (7,73)	12,99 (7,02)
Dorso-flexão plantar do tornozelo (°):		13,19 (13,65)	13,33 (11,87)
Progressão do pé (°):		14,04 (8,80)	10,64 (5,68)
**GDI**	> 100	73,23* (14,50)	80,45* (17,05)

**Abreviações:** DP, desvio padrão; GDI, gait deviation index; GPS, gait profile score; GVS, gait variable score. **Notas:** * valor de p < 0,05 na comparação com o valor padrão.

Na avaliação dos ângulos estáticos ( Tabela 2 ), os quadris apresentaram média de flexão acima do valor de normalidade ( Fig. 3 ). O arco de movimento dos joelhos também demonstrou importantes alterações, chegando a um valor individual máximo de 56.8° de flexão no joelho direito e de 48,9° no esquerdo. A maioria dos pacientes (54,5%) apresentou um padrão de marcha com aumento da flexão e adução dos quadris e flexão dos joelhos.

**Fig. 3:** ( **a** ) Paciente durante o exame estático (standing) com protocolo Helen Hayes MM no laboratório de marcha. ( **b** ) Reconstrução 3D no BTS SMART-Clinic. Notar o padrão em flexão dos quadris.

A média do GPS dos pacientes apresentou imp ortante desvio do parâmetro de normalidade. Em relação ao *Gait Variable Score* (GVS), a flexo-extensão e a rotação dos quadris, o arco de movimento dos joelhos e tornozelos e o ângulo de progressão do pé foram os valores médios mais elevados, sendo os maiores contribuintes para elevação do GPS destes pacientes. Todos os pacientes apresentaram GDI abaixo de 100, com menor valor individual sendo 59.67 ( Tabela 2 , Fig. 4 ).

**Fig. 4:** Gráficos do BTS motion analysys de diferentes pacientes do estudo. Notar o desvio da curva dos membros inferiores em relação ao parâmetro de normalidade em todas as imagens.

Sobre os dados de EMG, foram obtidas as atividades dos retos femorais, tíbias anteriores, gastrocnêmios (cabeça medial) e semitendinosos de 10 pacientes (um dos pacientes decidiu realizar o exame sem EMG).

Nossos resultados demostraram intervalos de atividade muscular que seguem aproximadamente o padrão habitual. No entanto, os resultados obtidos demostraram grande variabilidade entre os pacientes. Diferentes modalidades de ativação foram captadas, estando presentes mesmo em passadas consecutivas de um mesmo indivíduo. Houve variações no número de ativações, no tempo de início e deslocamento do sinal. Os músculos mais ativados durante o ciclo de marcha foram o tibial anterior (mais ativo em 60% dos pacientes) e o gastrocnêmio. Foi notada assimetria na ativação muscular no comparativo entre os membros em um mesmo paciente em 10% dos casos.

## Discussão

As deformidades esqueléticas são características marcantes presentes nos diferentes tipos de MPS. Defeitos angulares, como valgo dos joelhos e tornozelos, displasia progressiva dos quadris, frouxidão/ hipermobilidade articular são exemplos de alterações que levam a disfunções na marcha destes pacientes, 14 dificultando a deambulação e o equilíbrio e causando maior dependência funcional nas atividades cotidianas. 15

Um estudo realizado por Matos et al. 16 com 19 pacientes com MPS mostrou que as disfunções da marcha são as principais queixas relatadas por este grupo, sendo consideradas uma das maiores causas de incapacidade funcional nesta população.

A mobilidade humana representa um requisito fundamental para uma adequada qualidade de vida. A habilidade de andar é um indicador de saúde geral, pois denota autonomia. 17 Como a caminhada é um requisito básico para muitas atividades diárias, a análise da marcha fornece informações importantes sobre a capacidade funcional das pessoas.

O padrão de marcha dos indivíduos tornou-se uma área de grande interesse. A análise da marcha é amplamente utilizada como meio para diagnosticar doenças, avaliação de planos terapêuticos e do prognóstico de diversas patologias. 18 Desvios do padrão típico da marcha são características frequentes em doenças neurológicas, musculares e esqueléticas. 19

Cada tipo de MPS tem uma ampla gama de manifestações clínicas. 4 20 Além disso, um mesmo subtipo pode apresentar padrões muito variáveis de anormalidades do sistema osteoarticular e, por conseguinte, apresentar padrões variados de marcha. A avaliação 3D da marcha nestes pacientes permite uma melhor compreensão da história natural da doença nos seus diferentes fenótipos.

A velocidade média e, principalmente, o comprimento da passada estavam abaixo do normal em nosso estudo, semelhante ao descrito por Salazar-Torres. 21 Tal achado indica maior custo metabólico durante a marcha, como demonstrado por Kimoto et al. 22

O tempo de apoio mostra correlação importante com a estabilidade e o equilíbrio. Valores elevados do tempo de apoio representam diminuição da capacidade de equilíbrio do paciente. 23 O tempo de apoio médio em nosso estudo foi normal, demostrando que estes pacientes apresentam boa estabilidade durante a marcha.

Outro aspecto importante observado foi a assimetria entre os valores obtidos para cada membro. Os parâmetros que mais apresentaram diferenças entre os membros durante a marcha foram o arco de movimento dos joelhos e o ângulo de progressão do pé. Também foi notada assimetria nos valores temporais e, em menor percentual, nos dados de EMG. Sabe-se que quanto maior a assimetria dos membros inferiores durante a marcha, maior é o seu consumo energético. 24

A maioria dos nossos pacientes apresentaram um padrão de flexão e adução dos quadris e flexão dos joelhos. As alterações destas articulações são bem descritas tanto na síndrome de Maroteaux-Lamy quanto na de Morquio. Sabe-se que, especialmente nos quadris e joelhos destes pacientes, pode haver comprometimento desde os primeiros anos de vida. 25

O GPS e o GDI são índices que representam um resumo da qualidade geral da marcha, facilitando a comparação entre a deambulação patológica e a normal. Massad et al. 26 relataram o GDI como medida confiável para avaliação da marcha mesmo em uma única sessão. Em nosso estudo, os valores de ambos mostraram importante desvio do padrão da normalidade.

Os dados da EMG demonstraram intervalos de atividade muscular que seguiam aproximadamente o modelo vigente. No entanto, cabe ressaltar a grande variabilidade de padrões desse teste entre os pacientes, semelhante ao apontado por Agostini et al. 27 Um fator limitante na avaliação destes dados no presente estudo é que 54,5% dos pacientes avaliados eram crianças, das quais 45,5% correspondem a uma faixa de 4 a 8 anos. Embora os indivíduos nessa faixa etária sejam considerados com uma deambulação madura, em seu estudo, Granata et al. 28 demonstraram que a variabilidade da EMG intrasessão em crianças de 6 a 8 anos é duas vezes maior que a dos adultos. Já Agostini et al. 27 afirmaram que a atividade desse exame pode variar drasticamente entre as crianças, sendo que menos de 50% apresentam padrão similar. A grande variabilidade em crianças pode indicar um controle de estabilização mais responsivo do que nos adultos. 27

Um estudo de Fleming et al. 29 avaliou o nível de funcionalidade em 15 crianças com MPS, utilizando a Classificação Internacional de Funcionalidade, Incapacidade e Saúde, relatando comprometimento leve das funções corporais e comprometimento grave para mobilidade das articulações e marcha, em linha com o apontado pelo nosso estudo.

A limitação deste estudo é o pequeno número de sujeitos. No campo das síndromes genéticas de depósito, que cursam com acometimento de diferentes sistemas, sabe-se que a pesquisa deve lidar com o problema da grande variabilidade entre os pacientes e, geralmente, um grande número de indivíduos deve ser incluído nestes estudos. Assim, é necessário o desenvolvimento de mais pesquisas, com uma amostra mais ampla de pacientes.

## Conclusão

Os pacientes apresentaram uma velocidade média e comprimento da passada diminuídos. Um padrão de marcha em flexão e adução dos quadris e flexão dos joelhos foi identificado na maioria dos casos. Tanto GPS quanto GDI mostraram desvio importante da normalidade. A análise dos dados EMG demonstrou intervalos de atividade muscular que seguem aproximadamente o modelo vigente, ressaltando-se a grande variabilidade de padrões de ativação muscular entre os pacientes.
